# Effects of circadian clock genes and environmental factors on cognitive aging in old adults in a Taiwanese population

**DOI:** 10.18632/oncotarget.15493

**Published:** 2017-02-16

**Authors:** Eugene Lin, Po-Hsiu Kuo, Yu-Li Liu, Albert C. Yang, Chung-Feng Kao, Shih-Jen Tsai

**Affiliations:** ^1^ Institute of Clinical Medical Science, China Medical University, Taichung, Taiwan; ^2^ Vita Genomics, Inc., Taipei, Taiwan; ^3^ TickleFish Systems Corporation, Seattle, WA, USA; ^4^ Department of Public Health, Institute of Epidemiology and Preventive Medicine, National Taiwan University, Taipei, Taiwan; ^5^ Center for Neuropsychiatric Research, National Health Research Institutes, Miaoli County, Taiwan; ^6^ Department of Psychiatry, Taipei Veterans General Hospital, Taipei, Taiwan; ^7^ Division of Psychiatry, National Yang-Ming University, Taipei, Taiwan; ^8^ Department of Agronomy, College of Agriculture & Natural Resources, National Chung Hsing University, Taichung, Taiwan

**Keywords:** circadian clock genes, circadian rhythms, cognitive aging, gene-gene and gene-environment interactions, single nucleotide polymorphisms, Gerotarget

## Abstract

Previous animal studies have indicated associations between circadian clock genes and cognitive impairment . In this study, we assessed whether 11 circadian clockgenes are associated with cognitive aging independently and/or through complex interactions in an old Taiwanese population. We also analyzed the interactions between environmental factors and these genes in influencing cognitive aging. A total of 634 Taiwanese subjects aged over 60 years from the Taiwan Biobank were analyzed. Mini-Mental State Examinations (MMSE) were administered to all subjects, and MMSE scores were used to evaluate cognitive function. Our data showed associations between cognitive aging and single nucleotide polymorphisms (SNPs) in 4 key circadian clock genes, *CLOCK* rs3749473 (*p* = 0.0017), *NPAS2* rs17655330 (*p* = 0.0013), *RORA* rs13329238 (*p* = 0.0009), and *RORB* rs10781247 (*p* = 7.9 × 10^−5^). We also found that interactions between *CLOCK* rs3749473, *NPAS2* rs17655330, *RORA* rs13329238, and *RORB* rs10781247 affected cognitive aging (*p* = 0.007). Finally, we investigated the influence of interactions between *CLOCK* rs3749473, *RORA* rs13329238, and *RORB* rs10781247 with environmental factors such as alcohol consumption, smoking status, physical activity, and social support on cognitive aging (*p* = 0.002 ∼ 0.01). Our study indicates that circadian clock genes such as the *CLOCK*, *NPAS2*, *RORA*, and *RORB* genes may contribute to the risk of cognitive aging independently as well as through gene-gene and gene-environment interactions.

## INTRODUCTION

Circadian rhythms are naturally recurring cycles that influence the timing of biological events such as sleep-wake cycles, hormone release, and energy metabolism [1]. The intracellular molecular machinery underlying circadian rhythms indicates that circadian oscillations are stimulated and maintained by a panel of core circadian clock genes, defined as genes whose protein products are necessary components for generating circadian rhythms within individual cells [2]. Core circadian clock genes include the aryl hydrocarbon receptor nuclear translocator like (*ARNTL*), clock circadian regulator (*CLOCK*), cryptochrome circadian clock 1 (*CRY1*), cryptochrome circadian clock 2 (*CRY2*), neuronal PAS domain protein 2 (*NPAS2*), nuclear receptor subfamily 1 group D member 1 (*NR1D1*), period circadian clock 1 (*PER1*), period circadian clock 2 (*PER2*), period circadian clock 3 (*PER3*), RAR related orphan receptor A (*RORA*), and RAR related orphan receptor B (*RORB*) genes [2]. In mammals, the molecular clock mechanism is presently viewed as a complicated interplay of transcriptional feedback regulatory loops involving various circadian clock genes that control and support circadian rhythms [3]. The key circadian clock gene *ARNTL* encodes the ARNTL protein, which is an elementary helix-loop-helix protein that forms heterodimers with either CLOCK or NPAS2, two other primary helix-loop-helix proteins [2]. The ARNTL/CLOCK heterodimer initiates the transcription of numerous target genes such as *PER1*, *PER2*, *PER3*, *CRY1*, and *CRY2* via E-box elements in the promoters of the target genes [2]. One after another, the resulting PER and CRY proteins inhibit further ARNTL/CLOCK transcriptional activity; a new cycle then proceeds due to the low level of ARNTL/CLOCK transcription to enhance the robustness of the oscillations based on the main loop, an additional feedback loop is formed with nuclear receptors such as NR1D1 (or Rev-erb alpha), RORA, and RORB, which initiate the circadian transcription of the *ARNTL* gene and thus contribute to the timing of the core clock machinery [3].

Previous works have shown that the circadian clock is involved in regulation of brain cognitive functions such as memory, mood, and adaptation to novelty [4–6]. Furthermore, several animal studies have indicated that impairment of memory and adaptation to novelty can result from a loss-of-function mutation in circadian clock genes, such as *Arntl* [7], *Clock* [7], *Cry1* [8], *Cry2* [8], *Npas2* [9], and *Per2* [10]. In addition, an age-associated decline in the activity of the circadian clock can contribute to cognitive aging or declines in multiple brain functions, such as sleep, mood, and memory, indicating that disturbed circadian rhythms may affect cognitive functions [5]. Therefore, alterations of the circadian clock gene system may play a causative role in cognitive decline in neurodegenerative diseases such as Alzheimer's diseases (AD) [11, 12]. Specifically, several recent association studies have indicated that single nucleotide polymorphisms (SNPs) within the *ARNTL* and *CLOCK* genes are associated with AD risk [13–16].

While several encouraging findings on the relationship between circadian clock genes and cognitive aging have emerged, to our knowledge, human data is scarce in terms of SNPs. Moreover, the interplay between circadian clock genes and environmental factors such as alcohol consumption, smoking status, physical activity, and social support, has not been comprehensively evaluated in previous association studies. Given that circadian rhythms and their relevant genes may play a key role in the development of cognitive aging, we hypothesized that core circadian clock genes may contribute to the etiology of cognitive aging independently and/or through complex interactions. The gene panel investigated here comprises the 11 aforementioned core circadian clock genes (Supplementary Table 1), namely the *ARNTL*, *CLOCK*, *CRY1*, *CRY2*, *NPAS2*, *NR1D1*, *PER1*, *PER2*, *PER3*, *RORA*, and *RORB* genes.

## RESULTS

Table 1 describes the demographic and clinical characteristics of the study population, comprised of 634 subjects. The median MMSE score was 27 and the interquartile range was 25–29.

**Table 1:** Demographic and clinical characteristics of study subjects

Characteristic	Overall
No. of subjects, *n*	634
Mean age ± SD, years	64.2±2.9
Male, *n* (%)/Female, *n* (%)	318 (50.2)/316 (49.8)
Married, *n*	520
Living alone, *n*	54
Any physical activity, *n*	404
Current alcohol drinker, *n*	35
Current smoker, *n*	41
High school graduate, *n*	376
MMSE score, median (IQR)	27 (25–29)

Abbreviations: IQR = interquartile range, MMSE = Mini-Mental State Examination, SD = standard deviation.

Data are presented as mean ± standard deviation

First, we investigated the association between cognitive aging and the 11 circadian clock genes mentioned above. Among the 644 SNPs assessed in this study (Supplementary Table 1), there were 74 SNPs in 8 of the circadian clock genes that demonstrated evidence of association (*P* < 0.05) with MMSE scores (Table 2). However, only the association of the *RORB* rs10781247 SNP with MMSE scores nearly reached significance after applying a Bonferroni correction (*P* < 0.05/644 = 7.8 × 10^−5^). As shown in Table 2, the *RORB* rs10781247 SNP indicated an association with MMSE scores among subjects after adjusting for covariates such as age, gender, and education in genetic models, including the additive model (*P* = 0.0021) and dominant model (*P* = 7.9 × 10^−5^).

**Table 2:** Linear regression models of associations between the MMSE scores and 8 selective circadian clock genes, which have an evidence of association (*P* < 0.05)

						Additive model			Recessive model			Dominant model		
Gene	CHR	SNP	A1	A2	MAF	BETA	SE	*P*	BETA	SE	*P*	BETA	SE	*P*
*ARNTL*	11	rs4757151	G	A	0.35	0.26	0.18	0.1604	0.22	0.35	0.5268	0.56	0.23	0.0148
*CLOCK*	4	rs3749473	T	C	0.09	0.90	0.47	0.0561	1.66	0.94	0.0799	0.88	0.28	**0.0017**
		rs11932595	G	A	0.07	1.68	0.81	0.0388	3.27	1.63	0.0455	0.74	0.31	0.0168
		rs62303728	A	G	0.07	1.80	1.00	0.0711	3.51	2.00	0.0795	0.65	0.31	0.0358
*CRY2*	11	rs4756035	T	C	0.46	0.18	0.16	0.2478	0.59	0.27	0.0291	-0.12	0.25	0.6310
*NPAS2*	2	rs57365275	A	G	0.31	-0.41	0.21	0.0454	-0.76	0.40	0.0544	-0.25	0.22	0.2653
		rs59005495	T	C	0.33	-0.32	0.19	0.0895	-0.74	0.36	0.0416	0.03	0.23	0.9033
		rs983287	G	A	0.24	0.18	0.24	0.4540	0.16	0.47	0.7383	0.46	0.23	0.0410
		rs17024926	T	C	0.41	0.27	0.16	0.0948	0.27	0.29	0.3433	0.48	0.24	0.0481
		rs12712084	T	C	0.32	-0.05	0.19	0.7940	-0.45	0.36	0.2059	0.54	0.23	0.0175
		rs1369481	T	C	0.25	-0.01	0.23	0.9660	-0.31	0.44	0.4793	0.57	0.22	0.0108
		rs17654772	A	G	0.07	-2.53	1.00	0.0116	-5.05	2.00	0.0117	-0.24	0.34	0.4785
		rs17655330	A	C	0.08	-3.21	0.99	**0.0013**	-6.42	1.99	**0.0013**	-0.05	0.32	0.8860
		rs73945847	T	C	0.49	0.27	0.16	0.0920	0.63	0.27	0.0186	0.08	0.26	0.7597
		rs4851390	G	A	0.49	0.25	0.16	0.1246	0.59	0.26	0.0242	0.05	0.26	0.8350
		rs12622050	G	A	0.31	-0.45	0.20	0.0282	-0.96	0.39	0.0138	-0.04	0.23	0.8766
		rs4851391	C	G	0.25	-0.47	0.24	0.0505	-1.01	0.47	0.0302	0.03	0.23	0.8826
		rs4851392	A	G	0.15	-0.75	0.36	0.0364	-1.53	0.71	0.0323	-0.04	0.25	0.8666
		rs2305159	A	C	0.20	-0.59	0.28	0.0335	-1.17	0.54	0.0320	-0.15	0.23	0.5173
		rs1542179	A	G	0.22	-0.62	0.27	0.0208	-1.21	0.53	0.0215	-0.21	0.23	0.3668
		rs1542178	A	G	0.18	-0.65	0.32	0.0452	-1.26	0.64	0.0505	-0.24	0.24	0.3095
		rs3768988	G	A	0.36	0.36	0.17	0.0389	0.50	0.33	0.1304	0.51	0.23	0.0258
		rs62152925	T	C	0.22	-0.62	0.26	0.0147	-1.31	0.50	**0.0092**	-0.01	0.23	0.9649
		rs3754680	C	T	0.23	0.81	0.34	0.0176	1.56	0.67	0.0202	0.24	0.24	0.3135
*PER2*	2	rs1972874	C	G	0.31	-0.33	0.19	0.0901	-0.44	0.37	0.2320	-0.47	0.23	0.0367
*PER3*	1	rs118049345	T	C	0.07	-2.01	0.82	0.0141	-4.02	1.63	0.0139	-0.14	0.33	0.6664
*RORA*	15	rs17237283	C	T	0.16	-0.83	0.37	0.0249	-1.67	0.73	0.0234	-0.10	0.24	0.6711
		rs11635975	G	A	0.18	-0.56	0.28	0.0509	-1.15	0.56	0.0423	-0.04	0.24	0.8712
		rs2553236	C	T	0.31	0.50	0.22	0.0221	1.09	0.42	0.0100	-0.01	0.23	0.9590
		rs117194204	A	G	0.10	1.19	0.54	0.0274	2.35	1.07	0.0288	0.27	0.29	0.3524
		rs13329238	C	A	0.22	-0.46	0.26	0.0744	-0.67	0.51	0.1918	-0.77	0.23	**0.0009**
		rs8041466	T	C	0.23	-0.47	0.23	0.0454	-0.89	0.46	0.0536	-0.26	0.23	0.2606
		rs12913890	G	C	0.38	-0.31	0.17	0.0722	-0.66	0.32	0.0370	-0.09	0.23	0.6889
		rs7182392	T	C	0.21	-0.59	0.26	0.0252	-1.03	0.52	0.0473	-0.52	0.23	0.0251
		rs4775309	A	G	0.46	-0.36	0.16	0.0258	-0.44	0.27	0.1065	-0.52	0.25	0.0385
		rs11631432	C	T	0.43	0.25	0.16	0.1201	0.21	0.28	0.4515	0.48	0.24	0.0453
		rs4775311	C	T	0.40	0.28	0.16	0.0818	0.28	0.29	0.3403	0.52	0.24	0.0280
		rs8039990	T	C	0.32	-0.48	0.19	0.0126	-0.93	0.37	0.0114	-0.24	0.23	0.2844
		rs8040450	C	G	0.34	-0.49	0.19	0.0107	-0.96	0.36	**0.0084**	-0.22	0.23	0.3337
		rs341389	A	G	0.32	-0.48	0.19	0.0129	-0.93	0.37	0.0116	-0.24	0.23	0.2854
		rs16943284	C	T	0.23	-0.15	0.24	0.5243	-0.53	0.47	0.2632	0.46	0.23	0.0444
		rs7172917	T	C	0.40	0.35	0.17	0.0467	0.50	0.32	0.1145	0.41	0.23	0.0803
		rs2140442	T	C	0.09	1.41	0.63	0.0261	2.92	1.27	0.0214	-0.52	0.31	0.0916
		rs11638929	C	T	0.37	0.36	0.18	0.0447	0.40	0.34	0.2468	0.65	0.23	**0.0044**
		rs17237563	T	C	0.28	0.59	0.23	**0.0094**	1.05	0.44	0.0184	0.44	0.22	0.0503
		rs1523530	A	T	0.28	0.46	0.23	0.0497	0.81	0.45	0.0756	0.34	0.23	0.1352
		rs17303341	C	T	0.24	0.67	0.25	**0.0085**	1.19	0.50	0.0169	0.49	0.23	0.0326
		rs75336871	A	G	0.24	0.66	0.26	0.0108	1.18	0.51	0.0202	0.47	0.23	0.0401
		rs4775349	C	T	0.44	-0.30	0.16	0.0554	-0.35	0.28	0.2157	-0.50	0.24	0.0341
		rs17303369	T	C	0.22	0.63	0.28	0.0223	1.19	0.55	0.0302	0.33	0.23	0.1499
		rs6494243	G	A	0.13	-0.17	0.33	0.6089	-0.20	0.66	0.7554	-0.55	0.26	0.0336
		rs2280595	A	T	0.17	0.83	0.40	0.0381	1.63	0.79	0.0398	0.17	0.24	0.4769
		rs4775350	C	T	0.23	0.64	0.27	0.0171	1.20	0.53	0.0229	0.33	0.23	0.1558
		rs782948	A	G	0.12	0.001	0.37	0.9972	0.13	0.74	0.8607	-0.52	0.26	0.0495
		rs12902142	C	T	0.48	0.07	0.16	0.6604	0.70	0.27	0.0102	-0.57	0.25	0.0209
		rs76824799	T	C	0.15	0.90	0.41	0.0287	1.69	0.82	0.0398	0.61	0.27	0.0215
		rs60094610	A	G	0.13	0.36	0.47	0.4514	0.87	0.95	0.3595	-0.53	0.25	0.0353
		rs1437535	T	C	0.38	0.16	0.17	0.3488	0.72	0.32	0.0241	-0.47	0.23	0.0457
		rs1437537	T	C	0.38	0.15	0.17	0.3834	0.68	0.32	0.0330	-0.45	0.23	0.0531
		rs76431303	T	C	0.13	0.44	0.50	0.3815	1.04	1.01	0.3020	-0.55	0.25	0.0308
		rs719006	T	A	0.32	-0.44	0.20	0.0282	-0.79	0.38	0.0383	-0.30	0.23	0.1833
		rs1160694	G	A	0.32	-0.42	0.20	0.0327	-0.76	0.38	0.0441	-0.29	0.22	0.1956
		rs1159814	T	C	0.31	-0.40	0.20	0.0457	-0.68	0.39	0.0762	-0.35	0.22	0.1173
		rs78512626	C	A	0.11	1.17	0.54	0.0302	2.32	1.07	0.0309	0.21	0.28	0.4586
		rs4238351	A	G	0.36	0.05	0.17	0.7917	-0.25	0.32	0.4405	0.51	0.23	0.0257
		rs117080246	T	G	0.09	1.22	0.54	0.0229	2.40	1.07	0.0250	0.42	0.30	0.1670
		rs4775368	T	C	0.42	-0.17	0.15	0.2627	-0.66	0.27	0.0156	0.27	0.24	0.2600
		rs146660446	C	T	0.10	1.35	0.50	**0.0072**	2.73	1.00	**0.0066**	0.08	0.29	0.7945
*RORB*	9	rs1018584	A	C	0.09	-2.01	0.99	0.0427	-4.02	1.98	0.0428	-0.11	0.29	0.7117
		rs1157358	T	C	0.08	-2.76	1.41	0.0504	-5.52	2.81	0.0497	0.01	0.34	0.9779
		rs2273975	A	G	0.10	-1.92	0.71	**0.0067**	-3.78	1.41	**0.0075**	-0.40	0.28	0.1563
		rs11144039	C	T	0.46	0.31	0.16	0.0502	0.29	0.27	0.2780	0.55	0.25	0.0280
		rs72614684	T	C	0.46	0.31	0.16	0.0518	0.28	0.27	0.3074	0.56	0.25	0.0254
		rs10781247	G	A	0.48	0.50	0.16	**0.0021**	0.32	0.27	0.2461	1.01	0.26	**7.9 × 10**^−5^

Abbreviations: A1 = minor allele, A2 = major allele, BETA = Beta coefficients, Chr = chromosome, MAF = minor allele frequency, MMSE = Mini-Mental State Examination, SE = standard error.

Analysis was obtained after adjustment for covariates including age, gender, and education.

Next, we identified a nominal association of MMSE scores with 10 SNPs, including *CLOCK* rs3749473, *NPAS2* (rs17655330, rs62152925), *RORA* (rs13329238, rs8040450, rs11638929, rs17237563, rs17303341, rs146660446), and *RORB* rs2273975 (Table 2). For further investigation in the subsequent analyses, we selected four key SNPs in four circadian clock genes with evidence of association, including *CLOCK* rs3749473 (*P* = 0.0017), *NPAS2* rs17655330 (*P* = 0.0013), *RORA* rs13329238 (*P* = 0.0009), and *RORB* rs10781247 (*P* = 7.9 × 10^−5^). The genotype frequency distributions of these four SNPs were in accordance with the Hardy–Weinberg equilibrium among the subjects (*P* = 0.400, 0.390, 0.301, and 0.378, respectively).

We then employed categorized MMSE scores as an outcome (normal: MMSE score ≥ 24; cognitive impairment: MMSE score < 24) for gene-gene and gene-environment analysis. First, generalized multifactor dimensionality reduction (GMDR) analysis was used to assess the impacts of combinations between the four key SNPs in cognitive aging, including age, gender, and education as covariates. Table 3 summarizes the results obtained from GMDR analysis for two-way up to four-way gene-gene interaction models with covariate adjustment. There were significant two-way models involving *NPAS2* rs17655330 and *RORB* rs10781247 (*P* = 0.003), *RORA* rs13329238 and *RORB* rs10781247 (*P* = 0.003), *NPAS2* rs17655330 and *RORA* rs13329238 (*P*= 0.004), and *CLOCK* rs3749473 and *RORA* rs13329238 (*P* = 0.042). These results indicate potential gene-gene interactions between *NPAS2* and *RORB*, *RORA* and *RORB*, *NPAS2* and *RORA*, and *CLOCK* and *RORA*, respectively, in influencing cognitive aging. Additionally, there were a three-way model (*P* = 0.001) and a four-way model (*P* = 0.007) indicating potential gene-gene interactions among *CLOCK*, *NPAS2*, *RORA*, and *RORB* in influencing cognitive aging.

**Table 3:** Gene-gene interaction models identified by the GMDR method with adjustment for age, gender, and education

Interaction model	Testing accuracy (%)	*P* value
*CLOCK* rs3749473, *NPAS2* rs17655330	54.06	0.109
*CLOCK* rs3749473, *RORA* rs13329238	56.19	0.042
*CLOCK* rs3749473, *RORB* rs10781247	54.34	0.153
*NPAS2* rs17655330, *RORA* rs13329238	58.45	0.004
*NPAS2* rs17655330, *RORB* rs10781247	59.34	0.003
*RORA* rs13329238, *RORB* rs10781247	59.64	0.003
*NPAS2* rs17655330, *RORA* rs13329238, *RORB* rs10781247	62.48	0.001
*CLOCK* rs3749473, *NPAS2* rs17655330, *RORA* rs13329238, *RORB* rs10781247	60.82	0.007

Abbreviations: GMDR = generalized multifactor dimensionality reduction.

P value was based on 1,000 permutations. Analysis was obtained after adjustment for covariates including age, gender, and education.

Table 4 shows the GMDR analysis of gene-environment interaction models in cognitive aging using age, gender, and education as covariates. There was a significant two-way model involving *CLOCK* rs3749473 and environmental factors, namely smoking (*P* = 0.004), alcohol consumption (*P* = 0.01), and social support (*P* = 0.011), indicating potential gene-environment interactions between *CLOCK* and environmental factors in influencing cognitive aging. Similarly, there was a significant two-way model involving *RORA* rs13329238 and environmental factors such as smoking (*P* = 0.01), alcohol consumption (*P* = 0.002), physical activity (*P* = 0.01), and social support (*P* = 0.002). Finally, there was a significant two-way model involving *RORB* rs10781247 and the environmental factors alcohol consumption (*P* = 0.005) and physical activity (*P* = 0.035). However, there was no significant two-way model involving *NPAS2* rs17655330 and environmental factors.

**Table 4:** Gene-environment interaction models identified by the GMDR method with adjustment for age, gender, and education

Interaction model	Testing accuracy (%)	*P* value
*CLOCK* rs3749473, smoking	56.96	0.004
*CLOCK* rs3749473, alcohol consumption	56.23	0.010
*CLOCK* rs3749473, physical activity	53.98	0.153
*CLOCK* rs3749473, social support	56.71	0.011
*NPAS2* rs17655330, smoking	44.48	0.979
*NPAS2* rs17655330, alcohol consumption	48.78	0.668
*NPAS2* rs17655330, physical activity	44.69	0.917
*NPAS2* rs17655330, social support	50.48	0.444
*RORA* rs13329238, smoking	57.17	0.010
*RORA* rs13329238, alcohol consumption	59.31	0.002
*RORA* rs13329238, physical activity	57.95	0.010
*RORA* rs13329238, social support	58.16	0.002
*RORB* rs10781247, smoking	55.67	0.062
*RORB* rs10781247, alcohol consumption	58.26	0.005
*RORB* rs10781247, physical activity	56.93	0.035
*RORB* rs10781247, social support	54.31	0.135

Abbreviations: GMDR = generalized multifactor dimensionality reduction.

P value was based on 1,000 permutations. Analysis was obtained after adjustment for covariates including age, gender, and education.

Finally, Table 5 demonstrates a summarized model of the associations between the MMSE scores and SNPs within the *CLOCK*, *NPAS2*, *RORA*, and *RORB* genes. Table 5 also presents a summarized model of gene-gene and gene-environment interactions among the *CLOCK*, *NPAS2*, *RORA*, and *RORB* genes.

**Table 5:** Summarized model of the relationship between the MMSE scores and 4 selective circadian clock genes as well as their gene-gene and gene-environment interactions

Model	*P*
*CLOCK* rs3749473 (Dominant)	0.0017
*NPAS2* rs17655330 (Additive)	0.0013
*NPAS2* rs17655330 (Recessive)	0.0013
*NPAS2* rs62152925 (Recessive)	0.0092
*RORA* rs13329238 (Dominant)	0.0009
*RORA* rs8040450 (Recessive)	0.0084
*RORA* rs11638929 (Dominant)	0.0044
*RORA* rs17237563 (Additive)	0.0094
*RORA* rs17303341 (Additive)	0.0085
*RORA* rs146660446 (Additive)	0.0072
*RORA* rs146660446 (Recessive)	0.0066
*RORB* rs2273975 (Additive)	0.0067
*RORB* rs2273975 (Recessive)	0.0075
*RORB* rs10781247 (Additive)	0.0021
*RORB* rs10781247 (Dominant)	7.9 × 10^−5^
*CLOCK* rs3749473, *RORA* rs13329238	0.042
*NPAS2* rs17655330, *RORA* rs13329238	0.004
*NPAS2* rs17655330, *RORB* rs10781247	0.003
*RORA* rs13329238, *RORB* rs10781247	0.003
*NPAS2* rs17655330, *RORA* rs13329238, *RORB* rs10781247	0.001
*CLOCK* rs3749473, *NPAS2* rs17655330, *RORA* rs13329238, *RORB* rs10781247	0.007
*CLOCK* rs3749473, smoking	0.004
*CLOCK* rs3749473, alcohol consumption	0.010
*CLOCK* rs3749473, social support	0.011
*RORA* rs13329238, smoking	0.010
*RORA* rs13329238, alcohol consumption	0.002
*RORA* rs13329238, physical activity	0.010
*RORA* rs13329238, social support	0.002
*RORB* rs10781247, alcohol consumption	0.005
*RORB* rs10781247, physical activity	0.035

Abbreviations: MMSE = Mini-Mental State Examination.

Analysis was obtained after adjustment for covariates including age, gender, and education.

## DISCUSSION

To our knowledge, our study is the first to date to assess whether 644 SNPs in 11 circadian clock genes are significantly associated with the risk of cognitive aging independently and/or through gene-gene interactions among old Taiwanese individuals. We also examined the relationship between these genes and environmental factors to investigate whether these genes confer a risk of cognitive aging according to its effect on gene-environment interactions. Here, we report for the first time that several SNPs of the circadian clock genes, including *CLOCK* rs3749473, *NPAS2* rs17655330, *RORA* rs13329238, and *RORB* rs10781247, may play an important role in the modulation of cognitive aging in old adults in a Taiwanese population. Notably, the association of the *RORB* rs10781247 SNP with MMSE scores nearly persisted after correcting for multiple testing (*P* < 7.8 × 10^−5^). Additionally, our data revealed that gene-gene interactions between *CLOCK, NPAS2, RORA*, and *RORB* may contribute to the etiology of cognitive aging. Our data also indicated that there were gene-environment interactions between *CLOCK, RORA*, and *RORB* with environmental factors such as alcohol consumption, smoking status, physical activity, and social support.

To our knowledge, our results are the first to raise the possibility that 42 SNPs in the *RORA* gene and 6 SNPs in the *RORB* gene may contribute to susceptibility to cognitive aging, especially the *RORA* SNPs rs13329238, rs8040450, rs11638929, rs17237563, rs17303341, rs146660446, and the *RORB* SNPs rs10781247 and rs2273975. The proteins encoded by *RORA* (located on chromosome 15q22.2) and *RORB* (located on chromosome 9q2) constitute a subfamily of nuclear hormone receptors [3]. The *RORA* and *RORB* genes have been implicated in the regulation of a wide variety of physiological processes, including circadian rhythm, immunity, cellular metabolism, embryonic development, and inflammatory responses [17–18]. By using psychometric tests of cognitive function, Ersland et al. found that there was a strong association between the *RORB* gene and a test of verbal intelligence [19]. Consistent with this notion, Acquaah-Mensah et al. reported that the expression of the *RORA* gene is distinctly up-regulated in the hippocampi of AD-affected postmortem human brains, a brain region key to memory and learning, indicating a potential link between *RORA* and AD [20]. In terms of brain cognitive functions such as mood, the *RORA* gene was reported to increase the risk of acquiring psychiatric and neurological disorders, including bipolar disorder [6], autism spectrum disorder [21], and post-traumatic stress disorder [22]. Similarly, the *RORB* gene was also previously linked to psychiatric and neurological disorders, such as bipolar disorder [6, 23–24] and schizophrenia [23], in independent association studies.

In addition, an intriguing finding was a positive association of cognitive aging with 19 SNPs in the *NPAS2* gene, especially rs17655330 and rs62152925. The *NPAS2* gene, located on chromosome 2q11.2, encodes a member of the basic helix-loop-helix family of transcription factors. The functions of the NPAS2 and CLOCK proteins are partially redundant [25]. The ARNTL/CLOCK and ARNTL/NPAS2 heterodimeric proteins bind to chromatin, resulting in the up-regulation of *CRY1*, *CRY2*, *PER1*, *PER2*, and *PER3* gene expression [5]. Based on a series of behavioral tests, Garcia et al. found that *Npas2*-deficient mice may have impaired brain function and that NPAS2 may activate the neuronal gene expression required for the acquisition of long-term memory, indicating its importance for the execution of complex cognitive tasks [9].

On another note, we also observed that there was an association of cognitive aging with 3 SNPs in the *CLOCK* gene, particularly *CLOCK* rs3749473. The *CLOCK* gene is located on chromosome 4q12 and encodes a basic helix-loop-helix protein that constitutes the ARNTL/CLOCK heterodimeric protein with ARNTL, another basic helix-loop-helix protein [26]. Evidence has also been reported for the association of three separate SNPs of the *CLOCK* gene (rs4580704, rs1554483, and 3111) with AD in Chinese populations [13–15]; however, these findings have not been replicated by other large AD genetics consortiums. Additionally, Kondratova et al. reported that mice with mutations in the *Clock* gene showed declines in cognitive performance, including increased rearing activity and impaired intersession habituation [7]. Taken together, these studies suggest the engagement of the CLOCK protein in essential processes for memory formation [7].

Remarkably, another intriguing finding was that we further inferred the epistatic effects between *CLOCK, NPAS2, RORA*, and *RORB* in influencing cognitive aging by using the GMDR approach. To our knowledge, no other study has been conducted to evaluate gene-gene interactions between these genes. The functional relevance of the interactive effects between *CLOCK, NPAS2, RORA*, and *RORB* on cognitive aging remains to be elucidated. At the molecular level, the circadian clock consists of an interlocking network of several positive and negative feedback loops [27]. While the positive control of the circadian oscillator in the core loop involves ARNTL, CLOCK, and NPAS2 proteins, the negative control mechanism in the core loop comprises CRY1, CRY2, PER1, PER2, and PER3 proteins [27]. Additionally, an accessory pathway involving the RORs and NR1D1 nuclear receptors further stimulates the core loop [27]. Previous animal studies characterizing *Clock*/*Clock* mutant mice demonstrated that CLOCK is not indispensable for circadian rhythms, suggesting that NPAS2, as a substitute for CLOCK, can contribute to circadian rhythms in the absence of CLOCK [25, 28]. By reporter gene and mutation analysis, Takeda et al. also suggested that the RORs nuclear receptors are involved in the circadian regulation of the transcription of circadian clock genes such as the *Arntl*, *Cry1*, *Nr1d1*, and *Per2* genes [27]. Furthermore, Lai et al. found that there were potential interaction effects among the *RORA*, *RORB*, and *NR1D1* genes associated with an increased risk of bipolar disorder by using the multifactor dimensionality reduction method [6]. These studies suggest that the possible mechanisms of joint actions between these genes may synergistically incorporate other relevant circadian clock genes.

In the GMDR analysis of gene-environment interactions, we tracked down the interplay between the *CLOCK* gene and environmental factors (namely smoking, alcohol consumption, and social support), between the *RORA* gene and environmental factors (namely smoking, alcohol consumption, physical activity, and social support), and between the *RORB* gene and environmental factors ( namely alcohol consumption and physical activity). This interplay may manifest itself functionally through epigenetic changes. In previous animal studies, circadian deregulation was shown to affect epigenetic parameters such as DNA methylation, histone modifications, and miRNAs, suggesting that epigenetic alterations might be linked with age-related cognitive decline [29–31]. It has also been pointed out that core circadian clock genes may activate epigenetic modifications which are involved in circadian rhythm dysfunction with advancing age [31].

In this study, we further found evidence of potential association (*P* < 0.05) between cognitive aging and four other genes, the *ARNTL*, *CRY2*, *PER2*, and *PER3* genes, which are located on chromosomes 11p15, 11p11.2, 2q37.3, and 1p36.23, respectively. The *ARNTL* gene encodes a basic helix-loop-helix protein that forms the ARNTL/CLOCK heterodimeric protein with CLOCK [26]. The *CRY2* gene encodes a flavin adenine dinucleotide-binding protein that stimulates the circadian clock [8]. The *PER2* and *PER3* genes are members of the Period family, which encodes components of the circadian rhythms of locomotor activity, metabolism, and feeding behavior [10]. An association of AD with the rs2278749 SNP in the *ARNTL* gene in a Chinese population was previously observed [16], although this biomarker has not been identified by other large AD genetics consortiums. Contrary to our findings, Pereira et al. found no association of AD with SNPs in the *PER2* and *PER3* genes in a Brazilian population [32]. To our knowledge, no other study has been conducted to evaluate cognitive aging with SNPs in the *CRY2* gene. It is worth mentioning that multiple potential defects underly the discordant results found among these studies, including sample size, sample deviation, environmental control, study design, covariate adjustment, phenotype definitions, population stratification, and various ethnicities [33–36].

Our analysis indicated no association of cognitive aging with three other genes, the *CRY1*, *NR1D1*, and *PER1* genes, which are located on chromosomes 12q23-q24.1, 17q11.2, and 17p13.1, respectively. Like the *CRY2* gene, the *CRY1* gene encodes a flavin adenine dinucleotide-binding protein that stimulates the circadian clock [8]. The *NR1D1* gene encodes a member of the nuclear receptor subfamily 1 that negatively activates the expression of core clock proteins [6]. The *PER1* gene is a member of the same Period family as the *PER2* and *PER3* genes, which encodes components of the circadian rhythms of locomotor activity, metabolism, and feeding behavior [10]. To our knowledge, no other study has been conducted to evaluate the association between cognitive aging and SNPs in the *CRY1*, *NR1D1*, and *PER1* genes.

The MMSE was chosen to evaluate cognitive function, because it is well-established and is the most widely used screening test of cognition. Nonetheless, MMSE reduces variability in the data because it has a floor effect in the oldest adults and a ceiling effect in healthy young adults [37]. The General Practitioner Assessment of Cognition (GPCOG) was also found to have psychometric properties similar to the MMSE, but the GPCOG needs to be further assessed for its potential cultural or language bias [38, 39]. As a visual test, the Cambridge Neuropsychological Test Automated Battery (CANTAB) is a computer-based cognitive assessment system that is language independent [40]. However, aspects of CANTAB still need to be validated, and only modest correlations between CANTAB and traditional neuropsychological tests such as MMSE and GPCOG have been demonstrated [41]. Additionally, a well-validated scale in cognitive performance is the Alzheimer's Disease Assessment Scale – Cognitive section (ADAS-Cog), where a four-point change on ADAS-Cog has been established as a clinically important change in cognition [42]. Unfortunately, the length of administration time makes ADAS-Cog unsuitable for clinical practice because ADAS-Cog takes around 40 minutes to perform [37].

This study has both strengths and limitations. The main weakness is that our observations require much further research to determine whether the present research findings are sustained in diverse ethnic groups [34, 43]. To support the statistical analysis results, it is also desirable to have some additional evidence of biological functions, because the reported SNPs that influence the expression of the genes of interest have been shown to be importantly enriched for association studies [44]. In future work, prospective clinical trials with other ethnic populations would provide a comprehensive evaluation of the associations and interactions of the investigated genes with cognitive aging [35, 45, 46]. On the other hand, a major strength of our study is that we employed environmental data, which provided the opportunity to study the interplay between the investigated genes and environmental factors.

## CONCLUSIONS

In conclusion, we conducted an extensive analysis of the association as well as gene-gene and gene-environment interactions of the circadian clock genes with cognitive aging in old Taiwanese subjects. Overall, results from the current study revealed that the *CLOCK, NPAS2, RORA*, and *RORB* genes may affect the prevalence of cognitive aging independently and/or through complex gene-gene and gene-environment interactions. Future independent replication studies with larger numbers of subjects will likely lead to further insights into the roles of the circadian clock genes described in this study.

## MATERIALS AND METHODS

### Study population

This study incorporated subjects from the Taiwan Biobank [47–49]. The study cohort consisted of 634 participants. Recruitment and sample collection procedures were approved by the Internal Review Board of the Taiwan Biobank before conducting the study. Each subject signed the approved informed consent form. All experiments were performed in accordance with relevant guidelines and regulations.

Education was defined based on whether or not high school was attended. Current alcohol drinker was defined as currently drinking 150 ml of alcohol per week for more than six months. Current smoker was defined as currently smoking for more than six months. Physical activity was defined by the amount of exercise activity exceeding three times and more than 30 minutes each time in a week. Social support was assessed based on marital status and whether or not the subject lived alone.

### Cognitive assessment

Global cognitive assessment was performed using the 30-point Mini-Mental State Examination (MMSE), which includes questions based on the five domains of orientation, registration, attention and calculation, recall, and language. We analyzed MMSE as a continuous outcome, as well as according to categories based on previously defined MMSE thresholds [37]: MMSE score ≥ 24 (normal) and MMSE score < 24 (cognitive impairment).

### Genotyping

DNA was isolated from blood samples using a QIAamp DNA blood kit following the manufacturer's instructions (Qiagen, Valencia, CA, USA). The quality of the isolated genomic DNA was evaluated using agarose gel electrophoresis, and the quantity was determined by spectrophotometry [50, 51]. SNP genotyping was carried out using the custom Taiwan BioBank chips and run on the Axiom Genome-Wide Array Plate System (Affymetrix, Santa Clara, CA, USA). The SNP panel consisted of variants from the following 11 core circadian clock genes: *ARNTL*, *CLOCK*, *CRY1*, *CRY2*, *NPAS2*, *NR1D1*, *PER1*, *PER2*, *PER3*, *RORA*, and *RORB*.

### Statistical analysis

In this study, we evaluated the association of the investigated SNP with MMSE scores by a general linear model using age, gender, and education as covariates [36, 33]. The genotype frequencies were assessed for Hardy-Weinberg equilibrium using a *X*^2^ goodness-of-fit test with 1 degree of freedom (i.e. the number of genotypes minus the number of alleles). In order to correct for multiple testing, we applied a conservative Bonferroni correction factor for the number of SNPs employed in the analysis. The criterion for significance was set at *P* < 0.05 for all tests. Data are presented as the mean ± standard deviation.

To investigate gene-gene and gene-environment interactions, we employed the generalized multifactor dimensionality reduction (GMDR) method [52]. We tested two-way up to four-way interactions using 10-fold cross-validation. The GMDR software provided some output parameters, including the testing accuracy and empirical P values, to assess each selected interaction. Moreover, we provided age, gender, and education as covariates for gene-gene and gene-environment interaction models in our interaction analyses. Permutation testing obtained empirical P values of prediction accuracy as a benchmark based on 1,000 shuffles.

## SUPPLEMENTARY MATERIALS TABLES


