# Supplementary material for: Effects of circadian clock genes and environmental factors on cognitive aging in old adults in a Taiwanese population
Source: Oncotarget. 2017 Feb 16;8(15):24088–98. doi: 10.18632/oncotarget.15493 (PMC5421829; doi:10.18632/oncotarget.15493)
Supplement: Supplementary file 1 [file oncotarget-08-24088-s001.doc]

**Supplementary Table 1.** Linear regression models of associations between the MMSE scores and 644 SNPs in 11 circadian clock genes.

| Gene | CHR | SNP | Allele 1 | Allele 2 | P (Additive) | P (Recessive) | P (Dominant) |
| --- | --- | --- | --- | --- | --- | --- | --- |
| *ARNTL* | 11 | rs1481892 | G | C | 0.9273 | 0.8777 | 0.9795 |
|  |  | rs7950226 | G | A | 0.4747 | 0.6070 | 0.4740 |
|  |  | rs7951393 | T | C | 0.5196 | 0.1549 | 0.5946 |
|  |  | rs72867496 | G | A | 0.7499 | 0.6204 | 0.3431 |
|  |  | rs10832020 | C | T | 0.6230 | 0.4452 | 0.5376 |
|  |  | rs6486120 | T | G | 0.6422 | 0.3782 | 0.1243 |
|  |  | rs11022761 | T | C | 0.5240 | 0.4818 | 0.5731 |
|  |  | rs4757143 | C | T | 0.7094 | 0.6616 | 0.5630 |
|  |  | rs10741616 | G | A | 0.5680 | 0.3023 | 0.6899 |
|  |  | rs11022762 | C | T | 0.4218 | 0.1219 | 0.3333 |
|  |  | rs10766076 | A | T | 0.8287 | 0.4907 | 0.2473 |
|  |  | rs12805304 | A | T | 0.3713 | 0.8812 | 0.0944 |
|  |  | rs9633835 | G | A | 0.2900 | 0.1816 | 0.8445 |
|  |  | rs34188368 | T | C | 0.6201 | 0.5430 | 0.3576 |
|  |  | rs28711392 | T | C | 0.1838 | 0.0839 | 0.9295 |
|  |  | rs10766077 | G | A | 0.2182 | 0.0989 | 0.8778 |
|  |  | rs7924734 | G | A | 0.3008 | 0.9270 | 0.0686 |
|  |  | rs11022769 | C | A | 0.3008 | 0.9056 | 0.0738 |
|  |  | rs16912743 | G | A | 0.6607 | 0.5904 | 0.7658 |
|  |  | rs6486121 | T | C | 0.3391 | 0.3517 | 0.5821 |
|  |  | rs7947951 | G | A | 0.5328 | 0.3690 | 0.7769 |
|  |  | rs16912751 | C | T | 0.9897 | 0.9171 | 0.6053 |
|  |  | rs1026071 | G | A | 0.8261 | 0.9231 | 0.6376 |
|  |  | rs34834014 | C | T | 0.5831 | 0.5822 | 0.9325 |
|  |  | rs11022775 | T | C | 0.4205 | 0.4068 | 0.5359 |
|  |  | rs34991502 | G | T | 0.7907 | 0.7580 | 0.7072 |
|  |  | rs1868049 | C | T | 0.4258 | 0.5000 | 0.5341 |
|  |  | rs3789327 | G | A | 0.1278 | 0.2062 | 0.1275 |
|  |  | rs11022778 | G | T | 0.1338 | 0.1573 | 0.1560 |
|  |  | rs4757151 | G | A | 0.1604 | 0.5268 | 0.0148 |
|  |  | rs75854041 | C | T | 0.4669 | 0.4198 | 0.1675 |
|  |  | rs74762146 | A | G | 0.8751 | 0.7541 | 0.1605 |
|  |  | rs72869173 | A | G | 0.5345 | 0.5287 | 0.8925 |
|  |  | rs11022779 | A | G | 0.6495 | 0.7543 | 0.0552 |
|  |  | rs969485 | A | G | 0.1299 | 0.1892 | 0.2446 |
|  |  |  |  |  |  |  |  |
| *CLOCK* | 4 | rs3749473 | T | C | 0.0561 | 0.0799 | 0.0017 |
|  |  | rs6832769 | G | A | 0.5686 | 0.4581 | 0.8819 |
|  |  | rs11932595 | G | A | 0.0388 | 0.0455 | 0.0168 |
|  |  | rs12642716 | G | A | 0.2454 | 0.5369 | 0.1417 |
|  |  | rs62303728 | A | G | 0.0711 | 0.0795 | 0.0358 |
|  |  | rs11133391 | T | C | 0.3090 | 0.5935 | 0.1911 |
|  |  | rs7673908 | G | A | 0.5977 | 0.4721 | 0.8316 |
|  |  |  |  |  |  |  |  |
| [*CRY1*](mailto:HCRTR@) | 12 | rs7303842 | A | G | 0.2318 | 0.2327 | 0.6714 |
|  |  | rs79487478 | A | G | 0.4104 | 0.4730 | 0.1909 |
|  |  | rs11113179 | T | C | 0.1646 | 0.1686 | 0.5808 |
|  |  | rs17038985 | A | G | 0.2395 | 0.2430 | 0.6518 |
|  |  |  |  |  |  |  |  |
| [*CRY2*](mailto:HCRTR@) | 11 | rs10838524 | G | A | 0.6913 | 0.6779 | 0.9225 |
|  |  | rs11605924 | C | A | 0.6516 | 0.6442 | 0.8816 |
|  |  | rs4756034 | G | A | 0.2016 | 0.1294 | 0.8134 |
|  |  | rs4756035 | T | C | 0.2478 | 0.0291 | 0.6310 |
|  |  | rs2292912 | G | C | 0.1821 | 0.1218 | 0.7532 |
|  |  | rs11038699 | G | A | 0.8182 | 0.7741 | 0.2319 |
|  |  | rs2292910 | C | A | 0.3983 | 0.3168 | 0.9824 |
|  |  |  |  |  |  |  |  |
| *NPAS2* | 2 | rs13390078 | A | G | 0.1369 | 0.1441 | 0.4285 |
|  |  | rs6542992 | T | G | 0.4026 | 0.8881 | 0.2162 |
|  |  | rs75656425 | C | T | 0.3790 | 0.3763 | 0.8390 |
|  |  | rs77568479 | C | T | 0.8543 | 0.9821 | 0.2933 |
|  |  | rs78801659 | A | G | 0.4090 | 0.4089 | 0.8466 |
|  |  | rs3896080 | T | C | 0.8915 | 0.6437 | 0.0919 |
|  |  | rs79218385 | C | T | 0.5012 | 0.5291 | 0.4461 |
|  |  | rs3860455 | T | G | 0.2066 | 0.3449 | 0.0934 |
|  |  | rs57365275 | A | G | 0.0454 | 0.0544 | 0.2653 |
|  |  | rs72627416 | A | G | 0.0798 | 0.1005 | 0.2653 |
|  |  | rs6542994 | A | G | 0.9562 | 0.7616 | 0.7146 |
|  |  | rs17699370 | A | C | 0.1803 | 0.2167 | 0.2079 |
|  |  | rs55982284 | G | A | 0.1075 | 0.1076 | 0.6899 |
|  |  | rs62156093 | G | A | 0.1090 | 0.1093 | 0.6675 |
|  |  | rs13012930 | A | G | 0.6617 | 0.6665 | 0.7880 |
|  |  | rs4611661 | C | T | 0.5002 | 0.6615 | 0.1137 |
|  |  | rs4377354 | G | A | 0.7740 | 0.8987 | 0.0761 |
|  |  | rs72816926 | C | T | 0.6575 | 0.6384 | 0.7208 |
|  |  | rs11691732 | C | G | 0.4415 | 0.6042 | 0.0957 |
|  |  | rs7582455 | T | C | 0.5829 | 0.8153 | 0.0532 |
|  |  | rs2309992 | A | G | 0.3127 | 0.2959 | 0.8095 |
|  |  | rs7598826 | A | G | 0.3069 | 0.2813 | 0.6594 |
|  |  | rs59005495 | T | C | 0.0895 | 0.0416 | 0.9033 |
|  |  | rs75803056 | T | C | 0.2391 | 0.2714 | 0.2691 |
|  |  | rs76376883 | A | G | 0.7343 | 0.7469 | 0.6467 |
|  |  | rs1811399 | C | A | 0.2575 | 0.4563 | 0.1288 |
|  |  | rs983287 | G | A | 0.4540 | 0.7383 | 0.0410 |
|  |  | rs2043534 | T | C | 0.9280 | 0.9571 | 0.8099 |
|  |  | rs6759386 | T | G | 0.3788 | 0.5915 | 0.0684 |
|  |  | rs930309 | T | A | 0.4080 | 0.5355 | 0.4434 |
|  |  | rs12472321 | T | C | 0.3409 | 0.5651 | 0.2193 |
|  |  | rs12476292 | A | G | 0.2473 | 0.3060 | 0.1383 |
|  |  | rs17024926 | T | C | 0.0948 | 0.3433 | 0.0481 |
|  |  | rs72627426 | G | A | 0.5176 | 0.3628 | 0.7390 |
|  |  | rs72627427 | T | C | 0.5285 | 0.3631 | 0.7086 |
|  |  | rs12712084 | T | C | 0.7940 | 0.2059 | 0.0175 |
|  |  | rs1369481 | T | C | 0.9660 | 0.4793 | 0.0108 |
|  |  | rs17654772 | A | G | 0.0116 | 0.0117 | 0.4785 |
|  |  | rs920086 | A | G | 0.3683 | 0.5499 | 0.3187 |
|  |  | rs11123853 | A | G | 0.2582 | 0.1150 | 0.9865 |
|  |  | rs34333438 | G | A | 0.2416 | 0.2373 | 0.9526 |
|  |  | rs72627430 | T | C | 0.3496 | 0.3800 | 0.2359 |
|  |  | rs7570190 | G | T | 0.3627 | 0.3757 | 0.3255 |
|  |  | rs12989454 | T | C | 0.0880 | 0.0893 | 0.4942 |
|  |  | rs13418893 | A | G | 0.5943 | 0.9033 | 0.3526 |
|  |  | rs11894322 | C | A | 0.5995 | 0.8290 | 0.1207 |
|  |  | rs11894370 | G | A | 0.7053 | 0.8636 | 0.2894 |
|  |  | rs356643 | G | A | 0.2315 | 0.6580 | 0.1231 |
|  |  | rs77985008 | T | C | 0.6128 | 0.6145 | 0.8460 |
|  |  | rs7602455 | T | C | 0.5243 | 0.5722 | 0.5867 |
|  |  | rs17655330 | A | C | 0.0013 | 0.0013 | 0.8860 |
|  |  | rs6725296 | A | G | 0.3752 | 0.4372 | 0.1152 |
|  |  | rs79320559 | A | G | 0.6123 | 0.6129 | 0.8563 |
|  |  | rs356652 | G | T | 0.5568 | 0.2420 | 0.2663 |
|  |  | rs3754674 | C | G | 0.7877 | 0.8067 | 0.8283 |
|  |  | rs3754675 | C | T | 0.5432 | 0.2001 | 0.2258 |
|  |  | rs3820786 | A | G | 0.5669 | 0.5648 | 0.8868 |
|  |  | rs13025524 | A | G | 0.8131 | 0.8139 | 0.9233 |
|  |  | rs3768984 | C | A | 0.9516 | 0.9436 | 0.9965 |
|  |  | rs17025005 | T | C | 0.8218 | 0.7457 | 0.6238 |
|  |  | rs7605434 | G | A | 0.8383 | 0.8088 | 0.3180 |
|  |  | rs13429998 | G | A | 0.5772 | 0.5827 | 0.6251 |
|  |  | rs4851384 | A | G | 0.1152 | 0.0851 | 0.5458 |
|  |  | rs3820787 | G | A | 0.9194 | 0.5791 | 0.2762 |
|  |  | rs4851386 | T | C | 0.4244 | 0.8349 | 0.1567 |
|  |  | rs73945847 | T | C | 0.0920 | 0.0186 | 0.7597 |
|  |  | rs4851390 | G | A | 0.1246 | 0.0242 | 0.8350 |
|  |  | rs882272 | A | G | 0.4001 | 0.4036 | 0.6662 |
|  |  | rs895520 | A | G | 0.4007 | 0.4041 | 0.6736 |
|  |  | rs6738097 | C | T | 0.3445 | 0.2298 | 0.2672 |
|  |  | rs12622050 | G | A | 0.0282 | 0.0138 | 0.8766 |
|  |  | rs17025078 | A | G | 0.9270 | 0.8367 | 0.2325 |
|  |  | rs4851391 | C | G | 0.0505 | 0.0302 | 0.8826 |
|  |  | rs3768985 | C | T | 0.0729 | 0.0639 | 0.3062 |
|  |  | rs4851392 | A | G | 0.0364 | 0.0323 | 0.8666 |
|  |  | rs2289950 | T | C | 0.0862 | 0.0714 | 0.3424 |
|  |  | rs4851393 | T | C | 0.2056 | 0.6452 | 0.0830 |
|  |  | rs1562313 | T | C | 0.3727 | 0.2468 | 0.3126 |
|  |  | rs2305160 | A | G | 0.0888 | 0.0859 | 0.7012 |
|  |  | rs2305159 | A | C | 0.0335 | 0.0320 | 0.5173 |
|  |  | rs1542179 | A | G | 0.0208 | 0.0215 | 0.3668 |
|  |  | rs1542178 | A | G | 0.0452 | 0.0505 | 0.3095 |
|  |  | rs3768988 | G | A | 0.0389 | 0.1304 | 0.0258 |
|  |  | rs62152925 | T | C | 0.0147 | 0.0092 | 0.9649 |
|  |  | rs75763901 | T | C | 0.2931 | 0.2958 | 0.7297 |
|  |  | rs75159075 | A | C | 0.2396 | 0.2330 | 0.7508 |
|  |  | rs2278728 | T | C | 0.0676 | 0.0714 | 0.4280 |
|  |  | rs2278727 | T | C | 0.2507 | 0.3548 | 0.2885 |
|  |  | rs6719533 | G | A | 0.1181 | 0.0661 | 0.4863 |
|  |  | rs3754677 | C | T | 0.9902 | 0.9919 | 0.9915 |
|  |  | rs3754678 | G | A | 0.9796 | 0.9900 | 0.9673 |
|  |  | rs3754680 | C | T | 0.0176 | 0.0202 | 0.3135 |
|  |  | rs3768990 | C | T | 0.7790 | 0.8676 | 0.7179 |
|  |  | rs9223 | T | C | 0.9394 | 0.8712 | 0.6840 |
|  |  | rs3739008 | T | C | 0.8158 | 0.8082 | 0.9711 |
|  |  |  |  |  |  |  |  |
| *NR1D1* | 17 | rs883871 | G | A | 0.1880 | 0.2134 | 0.4074 |
|  |  | rs2071427 | C | T | 0.0595 | 0.0730 | 0.2262 |
|  |  | rs2269457 | T | C | 0.1614 | 0.1662 | 0.4111 |
|  |  | rs12941497 | G | A | 0.1912 | 0.2134 | 0.4007 |
|  |  | rs939347 | G | A | 0.1948 | 0.2211 | 0.3974 |
|  |  | rs2071570 | C | A | 0.2114 | 0.2553 | 0.3833 |
|  |  |  |  |  |  |  |  |
| *PER1* | 17 | rs2304911 | G | A | 0.3144 | 0.1743 | 0.1546 |
|  |  |  |  |  |  |  |  |
| *PER2* | 2 | rs934945 | T | C | 0.4677 | 0.7249 | 0.0564 |
|  |  | rs6431590 | G | A | 0.2275 | 0.3893 | 0.1354 |
|  |  | rs2304669 | C | T | 0.7280 | 0.7017 | 0.5181 |
|  |  | rs3739064 | G | A | 0.7972 | 0.7612 | 0.6818 |
|  |  | rs1972874 | C | G | 0.0901 | 0.2320 | 0.0367 |
|  |  | rs2304677 | T | C | 0.4016 | 0.3717 | 0.3897 |
|  |  | rs2304674 | G | A | 0.2702 | 0.3838 | 0.1727 |
|  |  | rs71426512 | T | C | 0.8813 | 0.8768 | 0.7483 |
|  |  | rs67860414 | T | G | 0.8805 | 0.9445 | 0.0956 |
|  |  |  |  |  |  |  |  |
| *PER3* | 1 | rs228727 | T | C | 0.8061 | 0.8853 | 0.5244 |
|  |  | rs11121023 | A | G | 0.1299 | 0.0759 | 0.2315 |
|  |  | rs75307902 | T | C | 0.0556 | 0.0566 | 0.5679 |
|  |  | rs10864315 | T | C | 0.3851 | 0.2722 | 0.3593 |
|  |  | rs117562183 | A | G | 0.1618 | 0.1627 | 0.6909 |
|  |  | rs228682 | C | T | 0.2772 | 0.2651 | 0.9626 |
|  |  | rs77567305 | C | T | 0.4451 | 0.4224 | 0.6133 |
|  |  | rs10746473 | G | A | 0.9373 | 0.8975 | 0.9846 |
|  |  | rs2797685 | C | T | 0.9556 | 0.9799 | 0.9050 |
|  |  | rs118049345 | T | C | 0.0141 | 0.0139 | 0.6664 |
|  |  | rs1773135 | A | G | 0.1686 | 0.1476 | 0.4913 |
|  |  | rs1689904 | C | T | 0.9727 | 0.9736 | 0.9268 |
|  |  | rs1773138 | T | C | 0.9477 | 0.9529 | 0.9617 |
|  |  | rs12563789 | G | A | 0.8102 | 0.8725 | 0.7103 |
|  |  |  |  |  |  |  |  |
| *RORA* | 15 | rs75461420 | A | C | 0.5181 | 0.5329 | 0.3637 |
|  |  | rs3743266 | C | T | 0.1110 | 0.1413 | 0.1434 |
|  |  | rs17270188 | A | G | 0.2541 | 0.1250 | 0.7890 |
|  |  | rs78280589 | G | A | 0.4328 | 0.4442 | 0.4486 |
|  |  | rs6494204 | C | T | 0.3836 | 0.3679 | 0.9277 |
|  |  | rs10438338 | C | T | 0.2989 | 0.3506 | 0.3110 |
|  |  | rs1866007 | G | A | 0.8020 | 0.7981 | 0.8989 |
|  |  | rs10519051 | G | A | 0.2526 | 0.2569 | 0.6297 |
|  |  | rs17237283 | C | T | 0.0249 | 0.0234 | 0.6711 |
|  |  | rs4594196 | C | T | 0.5741 | 0.6501 | 0.3243 |
|  |  | rs4774367 | G | C | 0.3768 | 0.3744 | 0.9741 |
|  |  | rs11635975 | G | A | 0.0509 | 0.0423 | 0.8712 |
|  |  | rs2028122 | A | G | 0.7638 | 0.7361 | 0.6492 |
|  |  | rs8033552 | A | G | 0.7317 | 0.8325 | 0.2734 |
|  |  | rs4775281 | C | A | 0.3572 | 0.3154 | 0.8758 |
|  |  | rs116861339 | C | T | 0.0823 | 0.0839 | 0.5260 |
|  |  | rs79360097 | C | T | 0.6423 | 0.6644 | 0.4254 |
|  |  | rs8041381 | G | A | 0.0776 | 0.0834 | 0.3048 |
|  |  | rs16942772 | T | G | 0.0885 | 0.0972 | 0.2750 |
|  |  | rs340002 | A | G | 0.7084 | 0.7312 | 0.0545 |
|  |  | rs11632600 | T | G | 0.2500 | 0.2850 | 0.3772 |
|  |  | rs11634234 | T | C | 0.5811 | 0.5147 | 0.9462 |
|  |  | rs340005 | G | A | 0.1206 | 0.1010 | 0.1828 |
|  |  | rs2289162 | A | T | 0.8680 | 0.9324 | 0.2814 |
|  |  | rs2289163 | C | A | 0.8951 | 0.9552 | 0.3188 |
|  |  | rs339969 | C | A | 0.1193 | 0.1018 | 0.2609 |
|  |  | rs12443044 | A | T | 0.2093 | 0.3864 | 0.2163 |
|  |  | rs72748739 | C | T | 0.8911 | 0.9557 | 0.2855 |
|  |  | rs340009 | A | C | 0.1839 | 0.3841 | 0.1677 |
|  |  | rs12591786 | T | C | 0.4727 | 0.4433 | 0.3121 |
|  |  | rs58306294 | T | C | 0.2913 | 0.3344 | 0.2115 |
|  |  | rs340021 | G | C | 0.5117 | 0.5826 | 0.1525 |
|  |  | rs340023 | C | T | 0.8953 | 0.5669 | 0.4701 |
|  |  | rs340026 | G | A | 0.5449 | 0.6026 | 0.3588 |
|  |  | rs3784611 | G | C | 0.5346 | 0.5988 | 0.3126 |
|  |  | rs3784610 | A | C | 0.3384 | 0.2142 | 0.9163 |
|  |  | rs28408562 | C | G | 0.1578 | 0.4129 | 0.1301 |
|  |  | rs28724570 | C | T | 0.1665 | 0.4442 | 0.1301 |
|  |  | rs75981965 | C | T | 0.9921 | 0.9916 | 0.9847 |
|  |  | rs1657792 | T | C | 0.8175 | 0.9442 | 0.2464 |
|  |  | rs11630262 | A | G | 0.1642 | 0.1741 | 0.1368 |
|  |  | rs78746013 | C | T | 0.4461 | 0.4468 | 0.8378 |
|  |  | rs75995412 | A | G | 0.7599 | 0.7414 | 0.7167 |
|  |  | rs2241794 | T | C | 0.4443 | 0.4326 | 0.4665 |
|  |  | rs80313728 | C | T | 0.4450 | 0.4315 | 0.3729 |
|  |  | rs339995 | A | G | 0.8156 | 0.3248 | 0.1791 |
|  |  | rs339996 | C | T | 0.2828 | 0.2624 | 0.9823 |
|  |  | rs17237318 | C | T | 0.0950 | 0.0759 | 0.8765 |
|  |  | rs339998 | T | C | 0.5018 | 0.4008 | 0.9683 |
|  |  | rs9630427 | C | T | 0.6248 | 0.3897 | 0.2622 |
|  |  | rs16942900 | T | C | 0.5624 | 0.6143 | 0.5252 |
|  |  | rs2433026 | C | G | 0.5528 | 0.6213 | 0.5828 |
|  |  | rs41356552 | G | A | 0.5704 | 0.3119 | 0.5495 |
|  |  | rs7166370 | C | G | 0.6413 | 0.7351 | 0.6369 |
|  |  | rs12437754 | A | G | 0.7390 | 0.5415 | 0.5589 |
|  |  | rs1657800 | C | T | 0.3799 | 0.4927 | 0.2733 |
|  |  | rs11629660 | C | A | 0.4863 | 0.6065 | 0.3322 |
|  |  | rs11629864 | C | G | 0.6980 | 0.7754 | 0.3244 |
|  |  | rs7172874 | C | T | 0.5506 | 0.7094 | 0.3212 |
|  |  | rs74800820 | G | A | 0.7463 | 0.7375 | 0.6678 |
|  |  | rs2553234 | T | C | 0.5676 | 0.6650 | 0.2922 |
|  |  | rs9920661 | C | A | 0.6021 | 0.6709 | 0.2942 |
|  |  | rs9920767 | A | G | 0.9695 | 0.9012 | 0.1330 |
|  |  | rs76105700 | G | A | 0.6640 | 0.6574 | 0.8561 |
|  |  | rs880626 | A | G | 0.2653 | 0.4659 | 0.2719 |
|  |  | rs880625 | G | A | 0.5418 | 0.3822 | 0.8542 |
|  |  | rs2553236 | C | T | 0.0221 | 0.0100 | 0.9590 |
|  |  | rs8040332 | A | T | 0.7626 | 0.7618 | 0.9831 |
|  |  | rs8042370 | T | C | 0.9438 | 0.6658 | 0.1900 |
|  |  | rs919000 | G | A | 0.8823 | 0.8040 | 0.7637 |
|  |  | rs999449 | A | C | 0.1415 | 0.1200 | 0.8988 |
|  |  | rs11629812 | A | G | 0.1069 | 0.0676 | 0.7758 |
|  |  | rs59558657 | T | C | 0.7852 | 0.7163 | 0.1488 |
|  |  | rs6494217 | A | G | 0.5527 | 0.3019 | 0.3309 |
|  |  | rs118138621 | T | G | 0.3322 | 0.3543 | 0.0559 |
|  |  | rs1425287 | A | G | 0.6700 | 0.7245 | 0.5973 |
|  |  | rs78164583 | A | C | 0.3492 | 0.3659 | 0.2063 |
|  |  | rs117194204 | A | G | 0.0274 | 0.0288 | 0.3524 |
|  |  | rs13329238 | C | A | 0.0744 | 0.1918 | 0.0009 |
|  |  | rs4774371 | A | G | 0.3152 | 0.3024 | 0.9424 |
|  |  | rs17237346 | T | C | 0.0753 | 0.0761 | 0.6026 |
|  |  | rs8027032 | T | C | 0.2967 | 0.2904 | 0.6809 |
|  |  | rs8038077 | C | T | 0.1510 | 0.2073 | 0.0743 |
|  |  | rs2433025 | A | G | 0.1309 | 0.1838 | 0.1670 |
|  |  | rs17303111 | T | C | 0.1499 | 0.1730 | 0.4042 |
|  |  | rs7173461 | C | G | 0.1098 | 0.1199 | 0.3694 |
|  |  | rs2414680 | A | G | 0.1585 | 0.1730 | 0.4600 |
|  |  | rs6494219 | C | T | 0.7177 | 0.6686 | 0.5939 |
|  |  | rs6494221 | C | A | 0.9595 | 0.9641 | 0.2262 |
|  |  | rs12899193 | T | C | 0.4413 | 0.5923 | 0.3914 |
|  |  | rs16943000 | G | A | 0.6568 | 0.6557 | 0.9885 |
|  |  | rs11071551 | G | C | 0.4198 | 0.4343 | 0.6328 |
|  |  | rs16943012 | G | C | 0.6016 | 0.5889 | 0.1861 |
|  |  | rs1834335 | T | C | 0.3204 | 0.3319 | 0.5939 |
|  |  | rs1820357 | T | G | 0.2452 | 0.1759 | 0.6269 |
|  |  | rs17237353 | A | G | 0.8525 | 0.8827 | 0.7018 |
|  |  | rs12591749 | C | T | 0.7129 | 0.7898 | 0.2308 |
|  |  | rs341413 | C | T | 0.4550 | 0.4772 | 0.5702 |
|  |  | rs35277300 | C | T | 0.7690 | 0.7036 | 0.9816 |
|  |  | rs17237367 | A | G | 0.5931 | 0.6293 | 0.6773 |
|  |  | rs78554936 | G | T | 0.9071 | 0.8709 | 0.2964 |
|  |  | rs7168905 | G | T | 0.9032 | 0.9094 | 0.8402 |
|  |  | rs9920962 | G | A | 0.4781 | 0.5333 | 0.6015 |
|  |  | rs76194223 | T | C | 0.7672 | 0.7323 | 0.2556 |
|  |  | rs4775292 | T | C | 0.9558 | 0.9763 | 0.2039 |
|  |  | rs7172011 | T | C | 0.6962 | 0.5530 | 0.5720 |
|  |  | rs1993471 | A | C | 0.9068 | 0.8755 | 0.3630 |
|  |  | rs17204367 | A | G | 0.5231 | 0.4327 | 0.8134 |
|  |  | rs17303153 | G | A | 0.7691 | 0.9728 | 0.5425 |
|  |  | rs1020729 | C | T | 0.5024 | 0.2877 | 0.8386 |
|  |  | rs58469372 | A | G | 0.7426 | 0.9806 | 0.4673 |
|  |  | rs1020730 | T | C | 0.4111 | 0.3588 | 0.8375 |
|  |  | rs17204402 | C | G | 0.6665 | 0.6024 | 0.4284 |
|  |  | rs961299 | A | G | 0.7432 | 0.7830 | 0.1552 |
|  |  | rs12900122 | T | C | 0.8094 | 0.7892 | 0.8308 |
|  |  | rs2279297 | C | T | 0.7589 | 0.7827 | 0.7082 |
|  |  | rs8025689 | C | G | 0.8357 | 0.8657 | 0.5966 |
|  |  | rs17204426 | G | T | 0.5814 | 0.5427 | 0.7996 |
|  |  | rs62002747 | C | T | 0.5836 | 0.5402 | 0.7556 |
|  |  | rs9302215 | C | T | 0.9912 | 0.7746 | 0.4883 |
|  |  | rs12591650 | A | G | 0.9658 | 0.4440 | 0.4709 |
|  |  | rs1482057 | A | C | 0.7500 | 0.6631 | 0.2184 |
|  |  | rs17204440 | C | A | 0.5195 | 0.5791 | 0.3653 |
|  |  | rs11639084 | T | C | 0.5309 | 0.5283 | 0.8943 |
|  |  | rs12594188 | C | T | 0.5880 | 0.6062 | 0.7461 |
|  |  | rs10519067 | A | G | 0.7051 | 0.7344 | 0.5282 |
|  |  | rs12438866 | C | T | 0.7051 | 0.8004 | 0.5372 |
|  |  | rs10519070 | T | C | 0.8130 | 0.7737 | 0.4691 |
|  |  | rs62002749 | A | G | 0.6756 | 0.6252 | 0.7329 |
|  |  | rs11071557 | C | T | 0.8198 | 0.8248 | 0.8966 |
|  |  | rs11071558 | G | A | 0.7816 | 0.7872 | 0.8777 |
|  |  | rs11071559 | T | C | 0.9003 | 0.9087 | 0.8963 |
|  |  | rs922782 | G | T | 0.8521 | 0.9363 | 0.5819 |
|  |  | rs922781 | C | G | 0.7014 | 0.7939 | 0.4956 |
|  |  | rs4774372 | C | T | 0.5799 | 0.5407 | 0.9713 |
|  |  | rs1963497 | A | C | 0.9592 | 0.9586 | 0.9998 |
|  |  | rs17270446 | G | C | 0.5519 | 0.6197 | 0.2814 |
|  |  | rs2899662 | T | C | 0.9936 | 0.9713 | 0.6549 |
|  |  | rs1680446 | T | C | 0.7823 | 0.7685 | 0.4808 |
|  |  | rs877228 | G | A | 0.8707 | 0.6295 | 0.6043 |
|  |  | rs4775297 | T | C | 0.2748 | 0.2503 | 0.9274 |
|  |  | rs16943117 | T | C | 0.9591 | 0.9683 | 0.5118 |
|  |  | rs12915776 | A | G | 0.7464 | 0.8177 | 0.4197 |
|  |  | rs341459 | C | T | 0.7074 | 0.6386 | 0.3229 |
|  |  | rs12593925 | T | C | 0.9208 | 0.5366 | 0.5686 |
|  |  | rs78498480 | C | G | 0.8902 | 0.8002 | 0.5987 |
|  |  | rs10162630 | A | G | 0.5626 | 0.6141 | 0.6341 |
|  |  | rs12591848 | A | C | 0.9087 | 0.6980 | 0.5178 |
|  |  | rs12440185 | T | C | 0.7482 | 0.7134 | 0.0611 |
|  |  | rs12902540 | T | C | 0.8675 | 0.9572 | 0.7167 |
|  |  | rs7162615 | A | G | 0.9123 | 0.9871 | 0.4659 |
|  |  | rs17270459 | T | C | 0.4040 | 0.4406 | 0.1492 |
|  |  | rs875339 | T | C | 0.8905 | 0.8372 | 0.7179 |
|  |  | rs62004360 | T | C | 0.4073 | 0.4406 | 0.1883 |
|  |  | rs341366 | A | G | 0.6915 | 0.1672 | 0.4030 |
|  |  | rs16943131 | C | T | 0.6024 | 0.5869 | 0.2071 |
|  |  | rs75866172 | A | G | 0.6026 | 0.5879 | 0.2171 |
|  |  | rs6494225 | G | T | 0.6007 | 0.5879 | 0.2821 |
|  |  | rs6494227 | C | A | 0.8346 | 0.7667 | 0.0772 |
|  |  | rs79610262 | T | C | 0.8307 | 0.7640 | 0.0820 |
|  |  | rs10519076 | C | G | 0.5525 | 0.5242 | 0.9469 |
|  |  | rs341373 | T | C | 0.4526 | 0.5069 | 0.1721 |
|  |  | rs78507043 | A | C | 0.4009 | 0.1640 | 0.9232 |
|  |  | rs10152719 | T | C | 0.6714 | 0.6852 | 0.8055 |
|  |  | rs341381 | G | A | 0.4104 | 0.4620 | 0.1187 |
|  |  | rs16943172 | T | C | 0.2437 | 0.2607 | 0.5546 |
|  |  | rs12439995 | G | C | 0.9763 | 0.9262 | 0.7680 |
|  |  | rs341392 | C | A | 0.3572 | 0.6802 | 0.1643 |
|  |  | rs6494229 | A | G | 0.6632 | 0.8313 | 0.5321 |
|  |  | rs8041061 | G | T | 0.9982 | 0.4238 | 0.2774 |
|  |  | rs8042149 | T | G | 0.9369 | 0.4773 | 0.4359 |
|  |  | rs4775301 | C | T | 0.2075 | 0.2809 | 0.3078 |
|  |  | rs6494230 | C | T | 0.3543 | 0.8798 | 0.1548 |
|  |  | rs11634976 | C | G | 0.5285 | 0.9604 | 0.2901 |
|  |  | rs8023252 | T | G | 0.3546 | 0.5140 | 0.3385 |
|  |  | rs341398 | G | A | 0.2384 | 0.4304 | 0.2423 |
|  |  | rs1224251 | G | A | 0.7183 | 0.7265 | 0.7392 |
|  |  | rs11630062 | C | T | 0.8984 | 0.9866 | 0.7908 |
|  |  | rs341403 | C | T | 0.5995 | 0.6151 | 0.7408 |
|  |  | rs12595623 | C | G | 0.7260 | 0.6922 | 0.9870 |
|  |  | rs11630227 | C | T | 0.6015 | 0.6803 | 0.6109 |
|  |  | rs10519080 | C | T | 0.4909 | 0.4622 | 0.9784 |
|  |  | rs341408 | T | C | 0.4091 | 0.3529 | 0.9427 |
|  |  | rs17204545 | G | T | 0.1695 | 0.1626 | 0.7686 |
|  |  | rs79409065 | A | C | 0.7475 | 0.7115 | 0.6043 |
|  |  | rs341411 | T | C | 0.2670 | 0.2567 | 0.6711 |
|  |  | rs75084363 | G | A | 0.3033 | 0.3474 | 0.2289 |
|  |  | rs11858268 | A | G | 0.1686 | 0.1523 | 0.2910 |
|  |  | rs7497885 | A | G | 0.2334 | 0.2029 | 0.8080 |
|  |  | rs2306502 | C | A | 0.1811 | 0.1762 | 0.7219 |
|  |  | rs10519085 | C | T | 0.1478 | 0.1686 | 0.3703 |
|  |  | rs8041466 | T | C | 0.0454 | 0.0536 | 0.2606 |
|  |  | rs12913890 | G | C | 0.0722 | 0.0370 | 0.6889 |
|  |  | rs72750668 | T | C | 0.7192 | 0.8166 | 0.6272 |
|  |  | rs77282013 | C | T | 0.0913 | 0.1168 | 0.1053 |
|  |  | rs1902618 | G | A | 0.2944 | 0.2784 | 0.9626 |
|  |  | rs341365 | A | G | 0.9065 | 0.4993 | 0.5933 |
|  |  | rs7182392 | T | C | 0.0252 | 0.0473 | 0.0251 |
|  |  | rs4775309 | A | G | 0.0258 | 0.1065 | 0.0385 |
|  |  | rs341387 | C | T | 0.2500 | 0.2199 | 0.7068 |
|  |  | rs11631432 | C | T | 0.1201 | 0.4515 | 0.0453 |
|  |  | rs4775311 | C | T | 0.0818 | 0.3403 | 0.0280 |
|  |  | rs8039990 | T | C | 0.0126 | 0.0114 | 0.2844 |
|  |  | rs8040450 | C | G | 0.0107 | 0.0084 | 0.3337 |
|  |  | rs341389 | A | G | 0.0129 | 0.0116 | 0.2854 |
|  |  | rs12907550 | T | C | 0.5763 | 0.5647 | 0.3667 |
|  |  | rs8036723 | A | G | 0.5994 | 0.3482 | 0.6604 |
|  |  | rs16943284 | C | T | 0.5243 | 0.2632 | 0.0444 |
|  |  | rs12915127 | T | C | 0.5154 | 0.5246 | 0.5532 |
|  |  | rs28692829 | T | C | 0.5735 | 0.4450 | 0.5250 |
|  |  | rs2414682 | C | T | 0.6371 | 0.5524 | 0.5011 |
|  |  | rs729977 | T | C | 0.5487 | 0.9030 | 0.1108 |
|  |  | rs7172917 | T | C | 0.0467 | 0.1145 | 0.0803 |
|  |  | rs4775313 | G | C | 0.2611 | 0.3498 | 0.0865 |
|  |  | rs4774376 | G | C | 0.0635 | 0.0516 | 0.4972 |
|  |  | rs35598844 | C | G | 0.8283 | 0.9214 | 0.5713 |
|  |  | rs7168782 | A | C | 0.6121 | 0.6216 | 0.8054 |
|  |  | rs117779544 | C | T | 0.8868 | 0.8595 | 0.0626 |
|  |  | rs8041087 | T | C | 0.6358 | 0.5473 | 0.3812 |
|  |  | rs2414686 | G | A | 0.5955 | 0.9487 | 0.2755 |
|  |  | rs877862 | G | A | 0.4318 | 0.3907 | 0.9363 |
|  |  | rs12904857 | A | G | 0.3488 | 0.3048 | 0.1384 |
|  |  | rs12910281 | C | T | 0.0950 | 0.3010 | 0.0720 |
|  |  | rs12909379 | A | G | 0.5505 | 0.9144 | 0.3426 |
|  |  | rs16943299 | A | G | 0.5972 | 0.5414 | 0.6134 |
|  |  | rs2899664 | A | G | 0.5939 | 0.6190 | 0.6972 |
|  |  | rs1054789 | T | A | 0.2738 | 0.3254 | 0.4169 |
|  |  | rs2062091 | C | T | 0.2075 | 0.2124 | 0.4684 |
|  |  | rs1384121 | A | C | 0.1225 | 0.1712 | 0.1163 |
|  |  | rs11855147 | C | T | 0.1729 | 0.1833 | 0.3387 |
|  |  | rs8027424 | C | A | 0.4507 | 0.2526 | 0.8164 |
|  |  | rs17237486 | T | C | 0.5176 | 0.4544 | 0.8961 |
|  |  | rs7162937 | C | G | 0.3874 | 0.4039 | 0.3409 |
|  |  | rs12148149 | C | T | 0.4004 | 0.4087 | 0.5803 |
|  |  | rs12901574 | A | C | 0.8679 | 0.5024 | 0.4029 |
|  |  | rs5813053 | C | N | 0.2781 | 0.1554 | 0.8785 |
|  |  | rs6494232 | A | G | 0.3175 | 0.4210 | 0.3914 |
|  |  | rs16943318 | A | G | 0.3676 | 0.3606 | 0.9528 |
|  |  | rs8028796 | C | T | 0.7628 | 0.7787 | 0.7755 |
|  |  | rs4775318 | T | A | 0.4668 | 0.5337 | 0.4974 |
|  |  | rs2062094 | T | C | 0.7485 | 0.7245 | 0.9889 |
|  |  | rs2062092 | C | T | 0.2620 | 0.2632 | 0.7301 |
|  |  | rs1482052 | T | G | 0.9056 | 0.9198 | 0.3202 |
|  |  | rs10220727 | T | C | 0.8394 | 0.7646 | 0.5253 |
|  |  | rs17303258 | G | A | 0.3226 | 0.3152 | 0.7886 |
|  |  | rs35715615 | G | A | 0.5383 | 0.7633 | 0.3369 |
|  |  | rs2279291 | T | G | 0.4253 | 0.7630 | 0.1320 |
|  |  | rs1482049 | C | T | 0.5210 | 0.9246 | 0.1466 |
|  |  | rs79995443 | C | A | 0.4754 | 0.4552 | 0.4923 |
|  |  | rs28705880 | T | G | 0.1723 | 0.4070 | 0.0984 |
|  |  | rs1351545 | T | C | 0.0953 | 0.0543 | 0.4640 |
|  |  | rs8034886 | A | G | 0.8266 | 0.4864 | 0.6343 |
|  |  | rs4775328 | T | C | 0.8039 | 0.8627 | 0.5017 |
|  |  | rs79271390 | C | T | 0.8935 | 0.9095 | 0.7280 |
|  |  | rs58413143 | C | A | 0.3665 | 0.2772 | 0.6362 |
|  |  | rs7176774 | T | C | 0.9513 | 0.9583 | 0.8180 |
|  |  | rs72752780 | C | A | 0.7338 | 0.6752 | 0.7247 |
|  |  | rs4335725 | T | C | 0.3070 | 0.2626 | 0.4945 |
|  |  | rs12903220 | T | C | 0.5871 | 0.5283 | 0.5691 |
|  |  | rs12593927 | G | C | 0.7493 | 0.9207 | 0.4551 |
|  |  | rs8029848 | G | A | 0.1319 | 0.1658 | 0.2656 |
|  |  | rs8034880 | G | A | 0.3562 | 0.4842 | 0.1472 |
|  |  | rs8034950 | C | T | 0.8881 | 0.6410 | 0.2947 |
|  |  | rs28575275 | A | G | 0.5883 | 0.8182 | 0.3283 |
|  |  | rs12912233 | T | C | 0.3219 | 0.2958 | 0.8709 |
|  |  | rs4775339 | A | G | 0.2339 | 0.2358 | 0.6632 |
|  |  | rs4775340 | A | G | 0.8777 | 0.9254 | 0.5781 |
|  |  | rs17237521 | T | C | 0.1293 | 0.1407 | 0.3788 |
|  |  | rs6494237 | C | T | 0.5818 | 0.6886 | 0.1161 |
|  |  | rs72752802 | C | A | 0.8601 | 0.7272 | 0.3328 |
|  |  | rs2140442 | T | C | 0.0261 | 0.0214 | 0.0916 |
|  |  | rs7168987 | T | C | 0.4890 | 0.4943 | 0.6926 |
|  |  | rs11631656 | G | A | 0.9545 | 0.9274 | 0.6791 |
|  |  | rs7176329 | T | C | 0.2146 | 0.2396 | 0.4475 |
|  |  | rs16943444 | A | G | 0.9808 | 0.9975 | 0.9523 |
|  |  | rs1467304 | C | T | 0.9580 | 0.9205 | 0.5697 |
|  |  | rs7174217 | T | C | 0.9467 | 0.7677 | 0.6650 |
|  |  | rs7171713 | T | C | 0.9345 | 0.9741 | 0.8249 |
|  |  | rs34299559 | N | G | 0.7878 | 0.6061 | 0.7090 |
|  |  | rs16943453 | G | T | 0.2336 | 0.2573 | 0.4752 |
|  |  | rs7174288 | A | G | 0.5461 | 0.4313 | 0.9807 |
|  |  | rs11637844 | G | A | 0.5337 | 0.3142 | 0.9479 |
|  |  | rs10519097 | T | C | 0.1968 | 0.1551 | 0.1891 |
|  |  | rs17204770 | C | T | 0.2017 | 0.1693 | 0.9202 |
|  |  | rs2030619 | G | T | 0.6320 | 0.6212 | 0.9062 |
|  |  | rs11638929 | C | T | 0.0447 | 0.2468 | 0.0044 |
|  |  | rs17237563 | T | C | 0.0094 | 0.0184 | 0.0503 |
|  |  | rs1523530 | A | T | 0.0497 | 0.0756 | 0.1352 |
|  |  | rs62005615 | A | C | 0.2686 | 0.2519 | 0.2266 |
|  |  | rs72625740 | C | T | 0.1021 | 0.0946 | 0.7502 |
|  |  | rs60257905 | T | C | 0.0641 | 0.0770 | 0.2182 |
|  |  | rs17237570 | C | T | 0.4263 | 0.2226 | 0.8949 |
|  |  | rs8040930 | G | A | 0.1570 | 0.1669 | 0.4642 |
|  |  | rs12898479 | G | A | 0.1661 | 0.1730 | 0.5198 |
|  |  | rs12592612 | C | T | 0.0911 | 0.1073 | 0.2129 |
|  |  | rs17303341 | C | T | 0.0085 | 0.0169 | 0.0326 |
|  |  | rs75336871 | A | G | 0.0108 | 0.0202 | 0.0401 |
|  |  | rs17303355 | C | G | 0.1782 | 0.1953 | 0.4337 |
|  |  | rs4775349 | C | T | 0.0554 | 0.2157 | 0.0341 |
|  |  | rs72625742 | T | C | 0.2445 | 0.3063 | 0.2846 |
|  |  | rs1403739 | G | A | 0.2838 | 0.3534 | 0.3012 |
|  |  | rs17303369 | T | C | 0.0223 | 0.0302 | 0.1499 |
|  |  | rs6494243 | G | A | 0.6089 | 0.7554 | 0.0336 |
|  |  | rs12438879 | G | T | 0.2894 | 0.2348 | 0.8948 |
|  |  | rs74687025 | G | A | 0.1382 | 0.1736 | 0.0568 |
|  |  | rs10519107 | C | G | 0.3612 | 0.2714 | 0.9148 |
|  |  | rs116919391 | A | C | 0.3290 | 0.3319 | 0.6724 |
|  |  | rs809736 | G | A | 0.2159 | 0.2593 | 0.1566 |
|  |  | rs2280595 | A | T | 0.0381 | 0.0398 | 0.4769 |
|  |  | rs4775350 | C | T | 0.0171 | 0.0229 | 0.1558 |
|  |  | rs1437549 | G | A | 0.5589 | 0.5630 | 0.7307 |
|  |  | rs4775351 | T | C | 0.5834 | 0.9356 | 0.2024 |
|  |  | rs7172342 | C | G | 0.3722 | 0.9095 | 0.1477 |
|  |  | rs4774384 | T | C | 0.2954 | 0.2832 | 0.7323 |
|  |  | rs782944 | A | C | 0.9985 | 0.6829 | 0.4260 |
|  |  | rs10519108 | C | G | 0.8940 | 0.8462 | 0.0940 |
|  |  | rs782948 | A | G | 0.9972 | 0.8607 | 0.0495 |
|  |  | rs2247306 | G | A | 0.3539 | 0.7527 | 0.2146 |
|  |  | rs12324535 | A | G | 0.8911 | 0.8914 | 0.6173 |
|  |  | rs873962 | A | G | 0.2475 | 0.4232 | 0.1593 |
|  |  | rs873961 | T | C | 0.5930 | 0.7194 | 0.2941 |
|  |  | rs8037669 | A | G | 0.3604 | 0.2771 | 0.9997 |
|  |  | rs7173460 | A | G | 0.5417 | 0.5478 | 0.7350 |
|  |  | rs6494246 | T | C | 0.8239 | 0.9922 | 0.2539 |
|  |  | rs78100524 | A | G | 0.7917 | 0.8653 | 0.0957 |
|  |  | rs782956 | G | T | 0.6979 | 0.5418 | 0.5206 |
|  |  | rs11632352 | T | C | 0.8775 | 0.9578 | 0.4528 |
|  |  | rs782903 | G | T | 0.3808 | 0.6283 | 0.2248 |
|  |  | rs16943579 | A | G | 0.4794 | 0.6398 | 0.0940 |
|  |  | rs11854619 | C | T | 0.1376 | 0.1364 | 0.9355 |
|  |  | rs12915672 | C | G | 0.2723 | 0.2898 | 0.5312 |
|  |  | rs12915830 | A | G | 0.0531 | 0.0561 | 0.2551 |
|  |  | rs708680 | G | C | 0.1292 | 0.1999 | 0.1603 |
|  |  | rs12903172 | C | T | 0.3599 | 0.2821 | 0.9513 |
|  |  | rs2689352 | C | T | 0.9606 | 0.8928 | 0.9235 |
|  |  | rs940222 | G | T | 0.5184 | 0.4261 | 0.9576 |
|  |  | rs62005642 | T | G | 0.5622 | 0.6131 | 0.3374 |
|  |  | rs7171287 | G | C | 0.8678 | 0.8808 | 0.8742 |
|  |  | rs1437551 | G | A | 0.5271 | 0.6023 | 0.1752 |
|  |  | rs77786240 | A | G | 0.1056 | 0.1072 | 0.5336 |
|  |  | rs782907 | A | G | 0.2383 | 0.4578 | 0.2134 |
|  |  | rs782908 | A | G | 0.8202 | 0.8017 | 0.7683 |
|  |  | rs4774386 | C | T | 0.3540 | 0.3633 | 0.6823 |
|  |  | rs893286 | C | A | 0.1132 | 0.1186 | 0.4260 |
|  |  | rs718911 | G | A | 0.1197 | 0.0893 | 0.8731 |
|  |  | rs76853459 | T | G | 0.7245 | 0.7049 | 0.4379 |
|  |  | rs12902142 | C | T | 0.6604 | 0.0102 | 0.0209 |
|  |  | rs4775356 | C | T | 0.0843 | 0.0748 | 0.9349 |
|  |  | rs782910 | A | G | 0.1068 | 0.0781 | 0.5560 |
|  |  | rs8035885 | A | G | 0.2158 | 0.1978 | 0.8365 |
|  |  | rs76824799 | T | C | 0.0287 | 0.0398 | 0.0215 |
|  |  | rs60094610 | A | G | 0.4514 | 0.3595 | 0.0353 |
|  |  | rs1437535 | T | C | 0.3488 | 0.0241 | 0.0457 |
|  |  | rs1437537 | T | C | 0.3834 | 0.0330 | 0.0531 |
|  |  | rs8042259 | T | C | 0.3785 | 0.1228 | 0.0774 |
|  |  | rs76431303 | T | C | 0.3815 | 0.3020 | 0.0308 |
|  |  | rs3803479 | A | G | 0.7124 | 0.9126 | 0.5929 |
|  |  | rs893288 | C | T | 0.4977 | 0.2042 | 0.1559 |
|  |  | rs78573683 | A | G | 0.3127 | 0.2429 | 0.3279 |
|  |  | rs13329643 | T | C | 0.4571 | 0.4540 | 0.9630 |
|  |  | rs782915 | A | G | 0.5864 | 0.5515 | 0.6490 |
|  |  | rs8024672 | A | C | 0.5853 | 0.5097 | 0.0890 |
|  |  | rs782919 | T | C | 0.1637 | 0.1473 | 0.7471 |
|  |  | rs782931 | G | A | 0.9270 | 0.5562 | 0.4253 |
|  |  | rs782933 | A | C | 0.1477 | 0.2144 | 0.1689 |
|  |  | rs782935 | C | T | 0.2522 | 0.3162 | 0.3907 |
|  |  | rs17303474 | C | T | 0.2033 | 0.2336 | 0.3759 |
|  |  | rs782937 | A | C | 0.7025 | 0.6256 | 0.8955 |
|  |  | rs16943672 | T | A | 0.2162 | 0.2314 | 0.0663 |
|  |  | rs4775360 | G | C | 0.5592 | 0.5084 | 0.9499 |
|  |  | rs7183595 | C | G | 0.0733 | 0.1004 | 0.2193 |
|  |  | rs11634887 | T | C | 0.2117 | 0.6949 | 0.0890 |
|  |  | rs719006 | T | A | 0.0282 | 0.0383 | 0.1833 |
|  |  | rs1160694 | G | A | 0.0327 | 0.0441 | 0.1956 |
|  |  | rs1159814 | T | C | 0.0457 | 0.0762 | 0.1173 |
|  |  | rs78512626 | C | A | 0.0302 | 0.0309 | 0.4586 |
|  |  | rs9788699 | T | C | 0.3471 | 0.5068 | 0.1085 |
|  |  | rs9788704 | T | C | 0.0792 | 0.1435 | 0.1466 |
|  |  | rs11071587 | G | A | 0.3468 | 0.5307 | 0.3408 |
|  |  | rs11071588 | T | G | 0.3601 | 0.4947 | 0.3889 |
|  |  | rs9788745 | G | A | 0.7205 | 0.5155 | 0.3676 |
|  |  | rs7163680 | G | T | 0.3503 | 0.0741 | 0.7866 |
|  |  | rs12900813 | T | C | 0.3029 | 0.0713 | 0.9015 |
|  |  | rs12900948 | T | C | 0.3330 | 0.0768 | 0.8401 |
|  |  | rs4238351 | A | G | 0.7917 | 0.4405 | 0.0257 |
|  |  | rs12592385 | T | C | 0.8420 | 0.6253 | 0.0768 |
|  |  | rs12900176 | T | C | 0.1980 | 0.0683 | 0.8933 |
|  |  | rs737112 | C | T | 0.4891 | 0.1192 | 0.6760 |
|  |  | rs17237759 | G | T | 0.1160 | 0.0891 | 0.4325 |
|  |  | rs17303509 | T | C | 0.3715 | 0.3307 | 0.7519 |
|  |  | rs1370433 | A | T | 0.2744 | 0.0550 | 0.2039 |
|  |  | rs117080246 | T | G | 0.0229 | 0.0250 | 0.1670 |
|  |  | rs17303523 | C | G | 0.4225 | 0.3006 | 0.0849 |
|  |  | rs17303530 | G | T | 0.5149 | 0.4003 | 0.1550 |
|  |  | rs4775368 | T | C | 0.2627 | 0.0156 | 0.2600 |
|  |  | rs11071590 | G | A | 0.6599 | 0.1426 | 0.4123 |
|  |  | rs11071591 | C | T | 0.6157 | 0.1513 | 0.5044 |
|  |  | rs7171405 | A | G | 0.5409 | 0.3776 | 0.2073 |
|  |  | rs4774388 | C | T | 0.4279 | 0.1813 | 0.3608 |
|  |  | rs4775370 | A | G | 0.5106 | 0.2280 | 0.3310 |
|  |  | rs1816624 | C | T | 0.5655 | 0.2949 | 0.4644 |
|  |  | rs4774390 | G | C | 0.4037 | 0.0633 | 0.2767 |
|  |  | rs4775371 | A | G | 0.6376 | 0.5815 | 0.4531 |
|  |  | rs1370431 | C | T | 0.8425 | 0.8343 | 0.9496 |
|  |  | rs12324086 | C | T | 0.6330 | 0.5660 | 0.8932 |
|  |  | rs17204952 | T | C | 0.3280 | 0.2836 | 0.5189 |
|  |  | rs17204959 | T | C | 0.6052 | 0.2770 | 0.7432 |
|  |  | rs12441507 | A | C | 0.8950 | 0.9692 | 0.1897 |
|  |  | rs1025676 | G | A | 0.2543 | 0.1828 | 0.3429 |
|  |  | rs55870008 | G | A | 0.8471 | 0.7061 | 0.6559 |
|  |  | rs7177846 | G | A | 0.5141 | 0.1725 | 0.8074 |
|  |  | rs2277557 | T | C | 0.9727 | 0.8499 | 0.1090 |
|  |  | rs10851691 | C | T | 0.7509 | 0.6750 | 0.9439 |
|  |  | rs10519116 | C | G | 0.4942 | 0.4229 | 0.3648 |
|  |  | rs726913 | A | G | 0.4726 | 0.2434 | 0.8312 |
|  |  | rs726955 | A | G | 0.6239 | 0.5614 | 0.4335 |
|  |  | rs2118326 | T | C | 0.8888 | 0.8948 | 0.5382 |
|  |  | rs34720147 | T | C | 0.2836 | 0.1585 | 0.8715 |
|  |  | rs4775374 | G | A | 0.1959 | 0.2298 | 0.1527 |
|  |  | rs17204973 | C | T | 0.2647 | 0.2902 | 0.4148 |
|  |  | rs1550226 | T | C | 0.2214 | 0.2262 | 0.6315 |
|  |  | rs11631786 | C | T | 0.2535 | 0.2828 | 0.3760 |
|  |  | rs11637553 | C | T | 0.7090 | 0.6344 | 0.6666 |
|  |  | rs12900971 | T | C | 0.5802 | 0.9354 | 0.2159 |
|  |  | rs146660446 | C | T | 0.0072 | 0.0066 | 0.7945 |
|  |  |  |  |  |  |  |  |
| *RORB* | 9 | rs17293191 | G | A | 0.3254 | 0.3286 | 0.5358 |
|  |  | rs4098048 | C | T | 0.6139 | 0.6243 | 0.4626 |
|  |  | rs13293006 | A | C | 0.3223 | 0.2877 | 0.3957 |
|  |  | rs28672222 | T | C | 0.6058 | 0.6177 | 0.4114 |
|  |  | rs1018584 | A | C | 0.0427 | 0.0428 | 0.7117 |
|  |  | rs4745330 | C | A | 0.1762 | 0.1559 | 0.4383 |
|  |  | rs7042950 | A | G | 0.8476 | 0.9012 | 0.6101 |
|  |  | rs10869418 | A | T | 0.8916 | 0.8692 | 0.9042 |
|  |  | rs17611535 | T | C | 0.5240 | 0.5221 | 0.9684 |
|  |  | rs7037043 | A | G | 0.2211 | 0.1901 | 0.4414 |
|  |  | rs75657768 | T | C | 0.4860 | 0.5658 | 0.3737 |
|  |  | rs972903 | C | T | 0.1633 | 0.0959 | 0.2067 |
|  |  | rs972902 | A | G | 0.1627 | 0.0949 | 0.2035 |
|  |  | rs17612113 | C | A | 0.5376 | 0.5235 | 0.5864 |
|  |  | rs1323354 | T | C | 0.9959 | 0.9974 | 0.9316 |
|  |  | rs62554058 | C | T | 0.1230 | 0.1044 | 0.3434 |
|  |  | rs67022110 | A | G | 0.7388 | 0.6445 | 0.1188 |
|  |  | rs10869430 | G | A | 0.3907 | 0.5444 | 0.3710 |
|  |  | rs75206074 | A | G | 0.8953 | 0.9180 | 0.2783 |
|  |  | rs1157358 | T | C | 0.0504 | 0.0497 | 0.9779 |
|  |  | rs11144029 | C | T | 0.7095 | 0.7329 | 0.7532 |
|  |  | rs3750420 | C | T | 0.2059 | 0.4657 | 0.1245 |
|  |  | rs1013078 | T | A | 0.6404 | 0.6519 | 0.7978 |
|  |  | rs10512037 | G | A | 0.0948 | 0.2853 | 0.0933 |
|  |  | rs11144032 | A | G | 0.4796 | 0.6855 | 0.3783 |
|  |  | rs2273975 | A | G | 0.0067 | 0.0075 | 0.1563 |
|  |  | rs1319551 | C | T | 0.1062 | 0.1070 | 0.6492 |
|  |  | rs10869433 | T | G | 0.7064 | 0.6795 | 0.8418 |
|  |  | rs11144039 | C | T | 0.0502 | 0.2780 | 0.0280 |
|  |  | rs72614684 | T | C | 0.0518 | 0.3074 | 0.0254 |
|  |  | rs59894901 | A | C | 0.6485 | 0.6969 | 0.2431 |
|  |  | rs10781247 | G | A | 0.0021 | 0.2461 | 7.93 x 10^-5^ |
|  |  | rs11144045 | G | T | 0.0928 | 0.1062 | 0.3626 |
|  |  | rs7865407 | G | T | 0.1507 | 0.1538 | 0.5774 |
|  |  | rs10869435 | A | T | 0.9461 | 0.7587 | 0.6568 |
|  |  | rs10869436 | G | A | 0.7188 | 0.9063 | 0.4684 |
|  |  | rs11144053 | C | G | 0.9118 | 0.9189 | 0.9009 |
|  |  | rs3818559 | A | C | 0.4839 | 0.5676 | 0.4993 |
|  |  | rs3793517 | T | G | 0.4412 | 0.3060 | 0.6151 |
|  |  | rs10521463 | G | T | 0.2167 | 0.1399 | 0.9341 |
|  |  | rs11144064 | T | C | 0.1676 | 0.1037 | 0.8772 |

Chr = chromosome, MMSE = Mini-Mental State Examination.

Analysis was obtained after adjustment for covariates including age, gender, and education.
